# The risk of COVID-19 in IBD patients is increased by urban living and is not influenced by disease activity or intravenous biologics

**DOI:** 10.3389/fimmu.2023.1243898

**Published:** 2023-08-28

**Authors:** Margaux Lelong, Régis Josien, Marianne Coste-Burel, Marie Rimbert, Céline Bressollette-Bodin, Stéphane Nancey, Guillaume Bouguen, Matthieu Allez, Mélanie Serrero, Ludovic Caillo, Cléa Rouillon, Pierre Blanc, David Laharie, Raphaël Olivier, Laurent Peyrin-Biroulet, Nina Dib, Astrid De Maissin, Céline Montuclard, Caroline Trang-Poisson, Fabienne Vavasseur, Géraldine Gallot, Mathilde Berthome, Cécile Braudeau, Justine Chevreuil, Arnaud Bourreille, Catherine Le Berre

**Affiliations:** ^1^Nantes Université, Centre Hospitalier Universitaire (CHU) Nantes, Institut des Maladies de l’Appareil Digestif (IMAD), Hépato-Gastro-Entérologie et Assistance Nutritionnelle, Institut national de la santé et de la recherche médicale (Inserm) CIC 1413, Nantes, France; ^2^Nantes Université, Centre Hospitalier Universitaire (CHU) Nantes, Laboratoire d’Immunologie Biologique, Centre d’ImmunoMonitorage Nantes-Atlantique (CIMNA), Nantes, France; ^3^Nantes Université, Institut national de la santé et de la recherche médicale (Inserm), Centre Hospitalier Universitaire (CHU) Nantes, CR2TI UMR 1064, Nantes, France; ^4^Nantes Université, Centre Hospitalier Universitaire (CHU) Nantes, Laboratoire de Virologie, Nantes, France; ^5^Department of Gastroenterology, Lyon-Sud Hospital, Hospices Civils de Lyon, Université Claude Bernard Lyon 1 and INSERM U1111-CIRI, Lyon, France; ^6^Centre Hospitalier Universitaire (CHU) and University of Rennes, INSERM, CIC1414, Institut NUMECAN (Nutrition Metabolism and Cancer), Rennes, France; ^7^Gastroenterology Department, Hôpital Saint-Louis, Assistance Publique-Hôpitaux de Paris (AP-HP), INSERM U1160, Université de Paris, Paris, France; ^8^Department of Gastroenterology, Centre Hospitalier Universitaire (CHU) Marseille, Marseille, France; ^9^Department of Gastroenterology, Nimes University Hospital, Nîmes, France; ^10^Department of Gastroenterology, Caen University Hospital, Caen, France; ^11^Service d’hépatogastroentérologie B, Centre Hospitalier Universitaire (CHU) Montpellier et Université Montpellier, Montpellier, France; ^12^Centre Hospitalier Universitaire (CHU) de Bordeaux, Centre Medico-chirurgical Magellan, Hôpital Haut-Lévêque, Gastroenterology department, Université de Bordeaux, INSERM CIC 1401, Bordeaux, France; ^13^Gastroenterology Department, University Hospital of Poitiers, Poitiers, France; ^14^Department of Gastroenterology and Inserm NGERE U1256, University Hospital of Nancy, University of Lorraine, Vandoeuvre-lès-Nancy, France; ^15^Department of HepatoGastroenterology, Angers University Hospital, Angers, France; ^16^Centre Hospitalier Départemental (CHD) La Roche Sur Yon, Department of Gastroenterology, La-Roche-sur-Yon, France; ^17^Department of Endoscopy and Gastroenterology, Valence Public Hospital, Valence, France; ^18^Nantes Université, Centre Hospitalier Universitaire (CHU) Nantes, Centre de ressources biologiques (CRB), Nantes, France

**Keywords:** Crohn’s disease, ulcerative colitis, infliximab, vedolizumab, SARS-CoV-2, trough levels

## Abstract

**Background:**

Patients with inflammatory bowel disease (IBD) may have a modified immune response to SARS-CoV-2. The objectives were to evaluate the prevalence of COVID-19 in patients treated with infliximab or vedolizumab, to analyze the factors associated with the infection, the impact of treatments and trough levels.

**Methods:**

Patients with IBD treated with intravenous biologics in 14 French centers were included between March and June 2020 and followed-up for 6 months. Blood samples were collected for serologies and trough levels. The analysis of factors associated with COVID-19 was conducted in a matched 1:1 case-control sub-study with positive patients.

**Results:**

In total, 1026 patients were included (74.9% infliximab). Over the follow-up period, 420 patients reported the occurrence of COVID-19 symptoms; 342 had been tested of whom 18 were positive. At the end of follow-up, 38 patients had a positive serology. Considering both nasal tests and serologies together, 46 patients (4.5%) had been infected. The risk of COVID-19 was related neither to the use of treatments (whatever the trough levels) nor to disease activity. Infections were more frequent when using public transport or living in flats in urban areas.

**Conclusions:**

The prevalence rate of COVID-19 in this IBD population treated with intravenous infliximab or vedolizumab was the same as the one in the French population before the start of the vaccination campaign. The risk was increased by urban living and was not influenced by disease activity or biologics. Sanitary barrier measures remain the best way to protect against SARS-CoV-2 in patients with IBD in biological therapy.

## 1 Introduction

Inflammatory bowel diseases (IBD), encompassing Crohn’s disease (CD) and ulcerative colitis (UC), are both characterized by chronic inflammation of the digestive tract due to dysregulation of the intestinal immune system. Although IBD do not significantly increase the risk of mortality (1), both CD and UC have been linked to an increased risk of death from infections, for multiple reasons, including chronic inflammation, undernutrition, and the therapies used that all have immunosuppressive properties, with the exception of 5-aminosalicylates (2–4).

Nowadays, more than 50% of patients are treated with immunosuppressants and/or biological therapies (5, 6). The risk of viral infections, in particular herpes viruses (7), is increased with thiopurines, which is less demonstrated with respiratory viruses such as the Influenzae virus (8). When using biologics, the risk of bacterial and opportunistic infections is greater, but this risk mainly concerns anti-tumor necrosis factor (TNF) agents, while ustekinumab and vedolizumab probably have a more favorable safety profile (9–11).

The infection by SARS-CoV-2 has raised questions about the management of patients with IBD. Since the beginning of the COVID-19 pandemic in December 2019, many studies have focused on the clinical factors associated with the risk of complications or death in general, such as older patients, particularly males, or those with co-morbidities including pulmonary, renal, cardiac, cerebrovascular or metabolic diseases (12–14). The link between drug-induced immunosuppression and severe COVID-19 is less clear (15, 16).

Several studies have reported a lower prevalence of SARS-CoV-2 infection in the IBD population, with a trend towards more frequent hospitalizations without increasing the risk of severe COVID-19 infection and admission in intensive care unit (17–21). Data in patients with IBD also tend to show a protective effect of anti-TNF agents (22–24), but there is still some uncertainty on the subject (25). Few studies studied the impact of trough levels on the risk of COVID-19. Data from the British CLARITY IBD study showed that the type of biologic agent did not impact the rates of PCR-confirmed SARS-CoV-2 infection, but seroprevalence rates were lower in infliximab- and adalimumab- than vedolizumab-treated patients. Interestingly, undetectable anti-TNF levels were associated with higher viral seropositivity rates, supporting a causal relationship. However, confounding factors, such as combination therapy with an immunosuppressant, have not been analyzed yet (26, 27).

Moreover, nowadays, vaccine safety and efficacy is a key question in the context of patients who have biological therapy, especially when seeing the rate of COVID-19 vaccine hesitancy in IBD patients (28, 29). Serologic response is good after vaccination in patients with IBD (30, 31), although lower with anti-TNF agents than with other treatments including vedolizumab (32). Real-word data have also proven the safety of COVID-19 vaccines in IBD patients (33, 34). Nevertheless, it is important to be able to provide patients with data regarding the risk of being infected by SARS-CoV-2 and the factors associated with this risk, especially when treated with biologics, outside any vaccination context.

The prospective MICI-SARS-CoV-2 study aimed at bringing more precision to these fundamental questions on a population of French patients with IBD being treated with intravenous infliximab or vedolizumab at day hospital before the start of the vaccination campaign. The objectives of this study were (i) to evaluate the prevalence of COVID-19 in a French cohort of patients with IBD treated with infliximab or vedolizumab during the first pandemic wave; (ii) to identify clinical and socio-demographic factors associated with the risk of COVID-19; (iii) to analyze the impact of biologics (with or without combination therapy with immunosuppressants) and their trough levels on the risk of being infected by SARS-CoV-2.

## 2 Materials and methods

### 2.1 Study design and setting

The MICI-SARS-CoV-2 study was a prospective multicenter study with an associated biological collection, starting during the first pandemic wave in France. This study was led in cooperation with the Groupe d’Etude Thérapeutique des Affections Inflammatoires du Tube Digestif (GETAID), of which all the 15 participating centers are members and are used to conduct collaborative research projects, allowing the rapid constitution of this large cohort.

The inclusion period started in March 2020 and lasted until June 2020 – the first two months of the study corresponding to the first lockdown period in France. Patients were recruited consecutively in the day hospital of each center. The follow-up period ended in January 2021 before the start of the vaccination campaign in France.

### 2.2 Study population

All patients with an established diagnosis of IBD (CD, UC or IBD-unclassified), aged over 18, treated with either intravenous (IV) infliximab or vedolizumab, could be included. No patient under subcutaneous biologic therapy was included in this study in order to have a homogeneous population of patients during this lockdown period – all included patients had to leave home and come to day hospital to receive their treatment. Non-inclusion criteria included patients under legal protection (guardianship, curatorship) or under safeguard of justice, insufficient command of French language, and contra-indications to infliximab or vedolizumab at baseline.

At baseline, patient information was given orally and in writing, then the physician collected patient’s oral non-opposition. Patients were followed-up for a period of 6 months at a rhythm depending on the interval between infliximab or vedolizumab infusions.

### 2.3 Questionnaire administration and data collection procedures

Clinical data were collected by the physician at baseline, including gender, type of IBD (CD, UC or IBD-unclassified), age at baseline, disease duration, body mass index (BMI) at baseline, smoking status, disease location and behavior according to the Montreal classification, history of perianal disease, presence of extra-intestinal manifestations, presence of comorbidities, history of intestinal resection, ongoing disease-related treatments, previous treatments used, disease activity index (Harvey Bradshaw Index [HBI] for CD, Mayo clinical sub-score for UC), and level of C-reactive protein (CRP) if available.

At baseline, patients filled-in a questionnaire regarding their lifestyle during the lockdown period, including type of professional activity (continuation of their usual activity, teleworking or stopping work), their family environment (number of people living at home, including those who continued their professional activities, as well as the number of children under 15 years of age), their type of accommodation (house or flat), their area of residence (city center, suburban area, rural area), and their means of transport to come to the day hospital (private car, public transport, other including taxi).

At each visit, patients filled-in another questionnaire on the occurrence of an intercurrent event due to their IBD, especially the need of corticosteroids between two infusions (self-medication was not excluded). An active disease during the follow-up period was defined by treatment intensification (addition of at least one medication, shortening of the interval between infusions, increase in the dose of infliximab infusion), change of biological therapy, steroids intake, hospitalization(s) or emergency consultation(s) with their general practitioner or gastroenterologist.

At each of their visit at day hospital, patients were also questioned about the occurrence of symptoms that could suggest COVID-19 during the interval since the last infusion (fever, cough, anosmia, myalgias, dyspnea, aches and pains, or other), and if so the performance of diagnostic tests (rapid antigen test, Polymerase Chain Reaction [PCR] test via nasal swab, or computed-tomography [CT] scan). It should be noted that the occurrence of symptoms suggestive of COVID-19 did not systematically result in testing in all patients. SARS-CoV-2 infection was considered as confirmed by a positive rapid antigen test and/or positive PCR test and/or a CT scan with characteristic lung involvement.

### 2.4 Blood sample collection procedures

After having obtained the subject’s written consent, blood samples were collected at each visit immediately before drug administration.

A dry blood tube of 10 mL was collected at each visit for all patients included, then prepared at 4°C and divided into 3 aliquots of serum of 1 mL frozen at – 80°C. These aliquots were used for SARS-CoV-2 antibody assays and dosage of infliximab or vedolizumab trough levels. Residual blood samples collected during the study were kept for new scientific interest. In 9 centers, 3 additional blood ethylenediaminetetraacetic acid (EDTA) tubes of 10 mL were collected at each visit, then prepared at room temperature for isolating peripheral blood mononuclear cells (PBMC) and frozen either at – 80°C or at – 130°C depending on the future immunological analyzes.

All samples from all centers were transferred to Nantes University Hospital using a specific frozen carrier, then integrated into the collection of human biological samples MICI-SARS-CoV-2 located in the Centre de Ressources Biologiques (CRB, BRIF: BB-0033-00040) of Nantes University Hospital.

### 2.5 Biological analysis

At the end of the follow-up, SARS-CoV-2 antibody assays were performed for all patients. For patients who were seropositive at the time of their last infusion, SARS-CoV-2 antibodies were assessed at each of their visit in order to determine the timing of seroconversion. SARS-CoV-2 antibodies were detected using the Elecsys^®^ Anti-SARS-CoV-2 S kit (Roche Diagnostics), an immunoassay for the qualitative detection of total antibodies (including IgG) directed against the spike protein receptor binding domain. In comparison with other similar tests, such as Abbott^®^ or Siemens^®^, the Elecsys^®^ kit has a good sensitivity and can detect more than 90% of positive samples 14 days after the onset of symptoms. It has also been showed to be well correlated to neutralizing antibodies results, with an area under the curve (AUC) between 0.959 and 0.987 in Receiver Operating Characteristic (ROC) curve analysis (35).

Residual drug levels were determined using the commercial Promonitor^®^ kits (Progenika Biopharma, Spain) supplied by GRIFOLS France SARL (Paris, France). They were performed at the time of seroconversion for seropositive patients, and at the end of follow-up for matched controls, in order to have comparable tubes already thawed once, then refrozen. The Promonitor^®^ ELISA tests are of the type “capture” for the measurement of infliximab, “sandwich” for the measurement of vedolizumab, “bridging” for the measurement of anti-drug antibodies. An enzymatic reaction using a chromogen (Tetramethylbenzidine [TMB]) then allows the quantification of the number of complexes (anti-infliximab/infliximab, anti-vedolizumab/vedolizumab) by evaluating the intensity of the colorimetric reaction spectrophotometrically and using a calibration curve. The signal obtained is proportional to the amount of infliximab, vedolizumab or anti-drug antibodies in the patient sample. The concentration measurement ranges are as follows: 0.3 to 14.4 µg/mL for infliximab trough levels, 3.5 to 54.8 μg/mL for vedolizumab trough levels, 5 to 1440 Arbitrary Unit (AU)/mL for anti-infliximab antibodies, and 27 to 300 AU/mL for anti-vedolizumab antibodies.

### 2.6 Statistical analysis

Statistics were performed using SAS 9.4 software.

#### 2.6.1 Descriptive statistics

Continuous variables are described using means and standard deviations and were tested using Student t-test for continuous variables. Categorical variables are described as raw counts and percentages and were tested using Chi-squared test (or Fisher’s exact test if statistically inappropriate). Missing values are systematically presented.

#### 2.6.2 Analysis of factors associated with SARS-CoV-2 infection

Due to the small number of patients tested positive for COVID-19 in the study population (confirmed either by positive rapid antigen test/PCR test/CT-scan during follow-up or by positive serology at the end of follow-up), a 1:1 case-control matching was performed according to several variables representing potential confounding factors in the risk of being infected by SARS-CoV-2 (age, gender, BMI, type of IBD, disease activity, use of an associated immunosuppressant). Matching by center could not be performed because there were too few patients per center for matching in addition to the other variables.

In this case-control sub-study, variables are described according to the COVID+/COVID– groups and in aggregate. Quantitative variables at baseline were compared between groups using a Student’s t test for paired data or a Mann-Whitney-Wilcoxon rank test for paired data. Binary categorical variables at baseline were compared between groups using a MacNemar test (for matched data). If more than two modalities, an exact symmetry test was performed to account for data matching. Variables measured at several visits (longitudinal follow-up) were analyzed using a mixed logistic model that took into account intra-patient correlation and matching by putting matching in random effect. In the model, the group, time (visit) effect and group*time interaction were analyzed.

### 2.7 Ethical considerations

This study was a non-interventional trial approved by the Comité de Protection des Personnes (CPP) Ile-de-France VI (institutional review board) on 30 March 2020 under the number 20.03.27.48341.

The blood samples were integrated into the collection of human biological samples attached to the « Hépato-Gastro-Entérologie » research program declared on 5 September 2011 under the number DC-2011-1399 and in the following amending declarations (DC-2012-1555; DC-2013-1832; DC2014-2206 and DC-2017-2987 currently pending) at the Ministry of Research and having obtained a favorable decision from the CPP Ouest IV on 7 April 2015. The consent form for this biological collection was validated by the CPP Ouest IV on 8 October 2020.

## 3 Results

**Figure 1:**
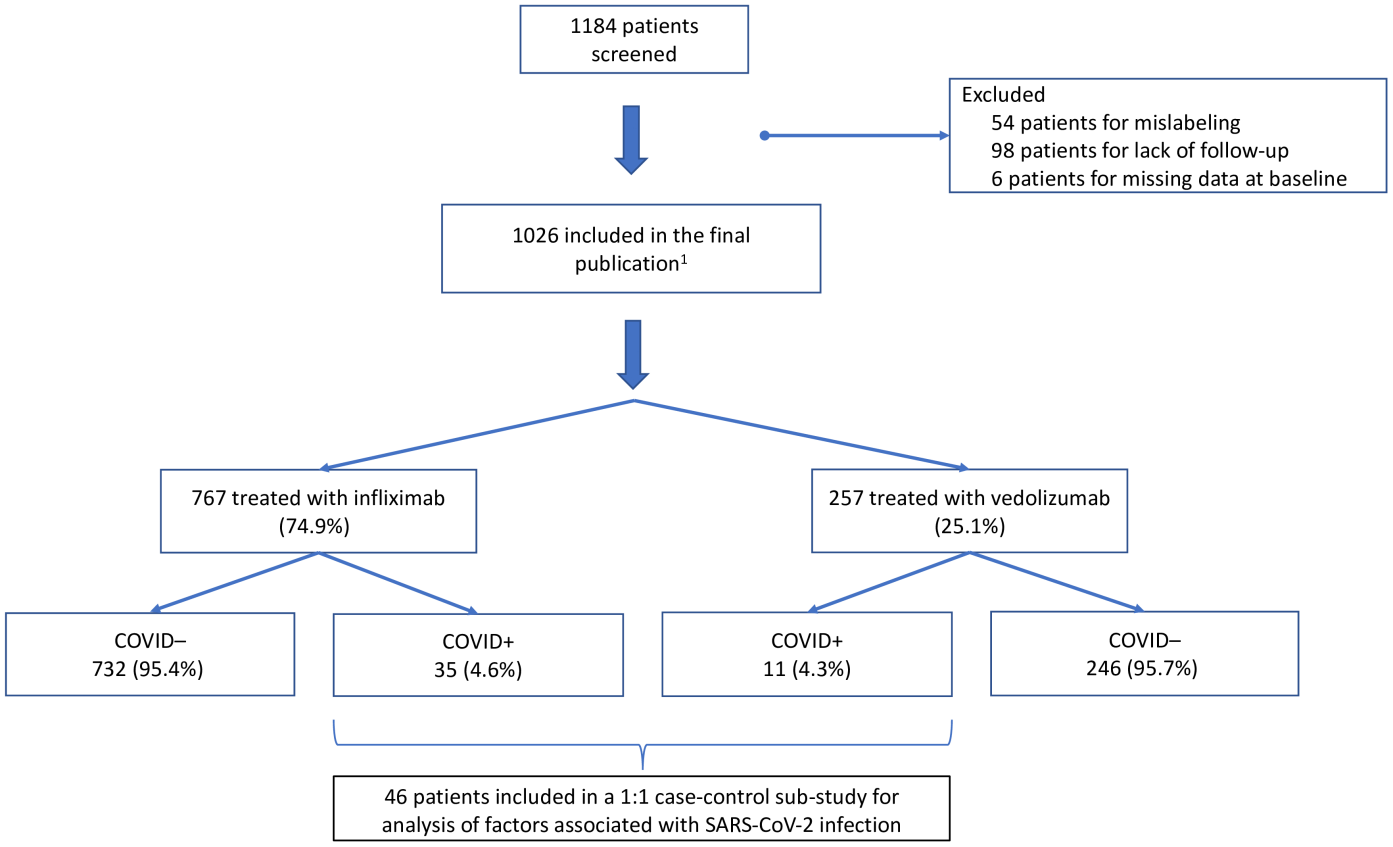
Flow chart of the study. ^1^: 2 missing data.

In total, 1184 patients were included in 17 centers. Patients from 2 centers had to be excluded due to lack of follow-up or mislabeling, and 6 patients were excluded due to missing data at baseline. Thus, serologies were performed on 1026 patients (**Figure 1**).

### 3.1 Baseline characteristics

Clinical characteristics of the study population at baseline are described in **Table 1**. More than 60.0% of patients had at least one extra-intestinal manifestation; the most frequent were rheumatologic manifestations, present in more than 11.2% of patients, particularly ankylosing spondylitis for 6.1% of them, and skin manifestations for 6.9%, especially psoriasis in 3.1% of cases. Digestive comorbidities including associated liver disease, primary sclerosing cholangitis or pancreatic disorders were present in 5.0% of cases. Regarding comorbidities that have been described to increase the risk of severe SARS-CoV-2 infection in the general population, 14.8% of patients were considered as obese, 5.4% had a pulmonary disease including chronic obstructive pulmonary disease (COPD) or asthma, 5.2% suffered from a vascular disease including hypertension, 2.3% had diabetes, 1.7% had a cardiac disease, 1.5% had cancer, 1.3% suffered from another autoimmune disease, and 0.9% had kidney failure.

**Table 1:** Clinical characteristics of the study population at baseline (n=1026).

	Total (n=1026)
Type of IBD, n (%)	
• CD	650 (63.4)
• UC	357 (34.8)
• IBD-unclassified	19 (1.9)
Gender, n (%)	
• Male	531 (51.8)
• Female	495 (48.2)
Age at baseline, mean (SD)	41.7 (15.4)
Disease duration^1^, mean (SD)	13.2 (9.3)
Active smoking^2^, n (%)	207 (21.5)
BMI (kg/m^2^)^3^, mean (SD)	25 (5.1)
• 30 ≤ BMI < 35, n (%)	109 (10.6)
• 35 ≤ BMI < 40, n (%)	32 (3.1)
• BMI > 40, n (%)	14 (1.4)
Disease location in CD^4^, n (%)	
• Ileal (L1)	152 (24.2)
• Colonic (L2)	141 (22.4)
• Ileocolonic (L3)	336 (53.4)
• Upper disease (L4)	62 (9.9)
• Perianal disease^5^	253 (39.9)
Disease location in UC^6^, n (%)	
• Proctitis (E1)	45 (13.2)
• Left-sided colitis (E2)	128 (37.4)
• Pancolitis (E3)	169 (49.4)
Disease behavior in CD^7^, n (%)	
• Inflammatory (B1)	309 (52.1)
• Stricturing (B2)	140 (23.6)
• Penetrating (B3)	144 (24.3)
Treatment at baseline, n (%)	
• Infliximab^8^	767 (74.9)
o Monotherapy	567 (73.9)
o Combination with thiopurines^9^	150 (19.6)
o Combination with methotrexate^10^	49 (6.4)
• Vedolizumab^11^	257 (25.1)
o Monotherapy	221 (85.3)
o Combination with thiopurines	26 (10.6)
o Combination with methotrexate^12^	8 (3.1)
• Corticosteroids^13^	53 (5.2)
Previous treatment^14^, n (%)	
• None	159 (15.8)
• Immunosuppresant	295 (29.3)
• Other biologic agent	174 (17.3)
• Combination therapy (all biotherapy included)	380 (37.7)
History of intestinal resection^15^, n (%)	251 (24.7)
Clinical scores	
• Harvey Bradshaw index^16^, mean (SD)	1.6 (2.8)
o < 4, n (%)	498 (81.8)
o 4-12, n (%)	103 (16.9)
o > 12, n (%)	8 (1.3)
• Mayo clinical sub-score^17^, mean (SD)	1.2 (1.9)
o < 2, n (%)	239 (71.5)
o 2-5, n (%)	77 (23.1)
o > 5, n (%)	18 (5.4)
C-reactive protein level, mean (SD)^18^	3.4 (8.6)
• > 5 mg/L, n (%)	215 (22.6)

CD, Crohn’s disease; IBD, Inflammatory bowel disease; SD, Standard deviation; UC, Ulcerative colitis.

Missing data: ^1^13; ^2^61; ^3^9; ^4^21; ^5^16; ^6^15; ^7^57; ^8^2; ^9^1; ^10^1; ^11^2; ^12^2; ^13^10; ^14^18; ^15^1; ^16^41;^17^23; ^18^32.

Socio-demographic data of the study population at baseline are described in **Table 2**.

**Table 2:** Socio-demographic characteristics of the study population at baseline (n=1026).

	Total (n=1026)
Professional status^1^, n (%)	
• Inactive (unemployed, retired, or sick leave)	605 (60.0)
• Active	403 (40.0)
o Teleworking	202 (20.0)
o Face-to-face	201 (19.9)
Family home, mean (SD)	
• Number of people living at home^2^	2.8 (1.4)
o Number of people continuing their professional activities^3^	1.2 (1.3)
• Number of children under 15^4^	0.8 (1.0)
Type of accommodation^5^, n (%)	
• Single family house	605 (59.8)
• Flat	405 (40.1)
Area of residence^6^, n (%)	
• City center	329 (32.9)
• Suburban area	286 (28.6)
• Rural area	386 (38.6)
Means of transport to come to the day hospital^7^, n (%)	
• Private car	791 (78.6)
• Public transport	95 (9.4)
• Other, including taxi	121 (12)

SD, Standard deviation.

Missing data: ^1^18; ^2^31; ^3^43; ^4^36; ^5^15; ^6^25; ^7^19.

### 3.2 Evolution of disease activity during follow-up

Over the 6 months of follow-up, 556 patients (54.2%) had an active disease defined by an HBI score >4 (n=159, 15.5%) or Mayo clinical sub-score >2 (n=105, 10.2%), and/or treatment intensification (addition of at least one medication or increase in the dose [n=341 (33.2%)], shortening of the interval between infusions [n=161, 15.7%], increase in the dose of infliximab infusion [n=75, 7.3%]), and/or change of biological therapy (n=19, 1.85%). Among them, 103 patients (10%) had received corticosteroids at least occasionally (self-medication was not excluded), 314 (30.6%) had visited their general practitioner, 37 patients (3.6%) had been hospitalized, and 33 patients (3.2%) had visited the emergency room for any reason during follow-up. The pandemic did not influence the adherence to intravenous biologics in this cohort because all patients kept their appointments in day hospital without any postponement.

### 3.3 SARS-CoV-2 outcomes during follow-up

All visits considered together, 420 patients (40.9%) reported the occurrence of symptoms suggestive of COVID-19 during the interval between two infusions. Although not specific, aches and pain were the most reported symptoms for 388 patients (37.8%). Cough was reported during follow-up by 225 patients (21.9%), dyspnea in 194 patients (18.9%). Anosmia was described in 120 patients (11.7%), and fever in 109 patients (10.6%). Of note, 150 patients (14.6%) reported other symptoms, notably digestive symptoms (diarrhea, abdominal pain).Over the 6 months of follow-up, 342 patients (33.3%) had been tested for SARS-CoV-2 infection, of whom 85 (24.9%) had been tested several times (twice or more). Most patients tested reported cough and dyspnea. The majority of patients (n=322, 94.2%) was tested by PCR test; rapid antigen test was performed in 19 patients (5.6%), of whom 2 also had a PCR test (10.5%); CT-scan was performed in 13 patients (3.8%), of whom 10 also had a PCR test (76.2%). Of the 342 tests, only 18 were positive (1 positive rapid antigen test, 17 positive PCR tests, no positive CT-scan). In those cases, the infusion of biologic was postponed by 14 days from the date of the positive test. There was no severe case of COVID-19. Only one patient had been hospitalized without requiring an admission in intensive care unit.

### 3.4 SARS-CoV-2 seroprevalence

**Table 3** summarizes the outcomes linked to SARS-CoV-2 infection in the study population. At the end of follow-up, 38 patients (3.7%) had anti-SARS-CoV-2 antibodies, of whom 28 (73.7%) never had a positive PCR test (25 had not been tested, 3 had been tested negative). Among the 3 patients who were tested negative by PCR with a positive serology at the end of follow-up, 2 of them were tested by PCR approximately at the same time as the serology was performed, but they had low levels of antibodies; the third patient had a negative PCR test 3 months before the detection of anti-SARS-CoV-2 antibodies which was strongly positive, probably reflecting a later contamination. Among the 18 patients who had been tested positive by nasal swab for SARS-CoV-2 during the follow-up, only 10 (55.6%) were seropositive at the end of follow-up.

**Table 3:** SARS-CoV-2 outcomes in the study population (n=1026).

	SARS-CoV-2 antibodies + (n=38)	SARS-CoV-2 antibodies – (n=988)
Antigen or PCR test + (n=18)	10 patients	8 patients
Antigen or PCR test – or not performed (n=1008)	28 patients^1^	980 patients

^1^25 patients had not been tested, 3 had been tested negative during the follow-up.

Considering both positive nasal tests and serologies together, 46 patients had been infected by SARS-CoV-2 during their follow-up in the first wave of the pandemic, i.e. 4.5% of the study cohort. None of these patients had any long-term sequelae of COVID-19.

### 3.5 Influence of clinical and socio-demographic factors on the risk of being infected by SARS-CoV-2

**Figure 2:**
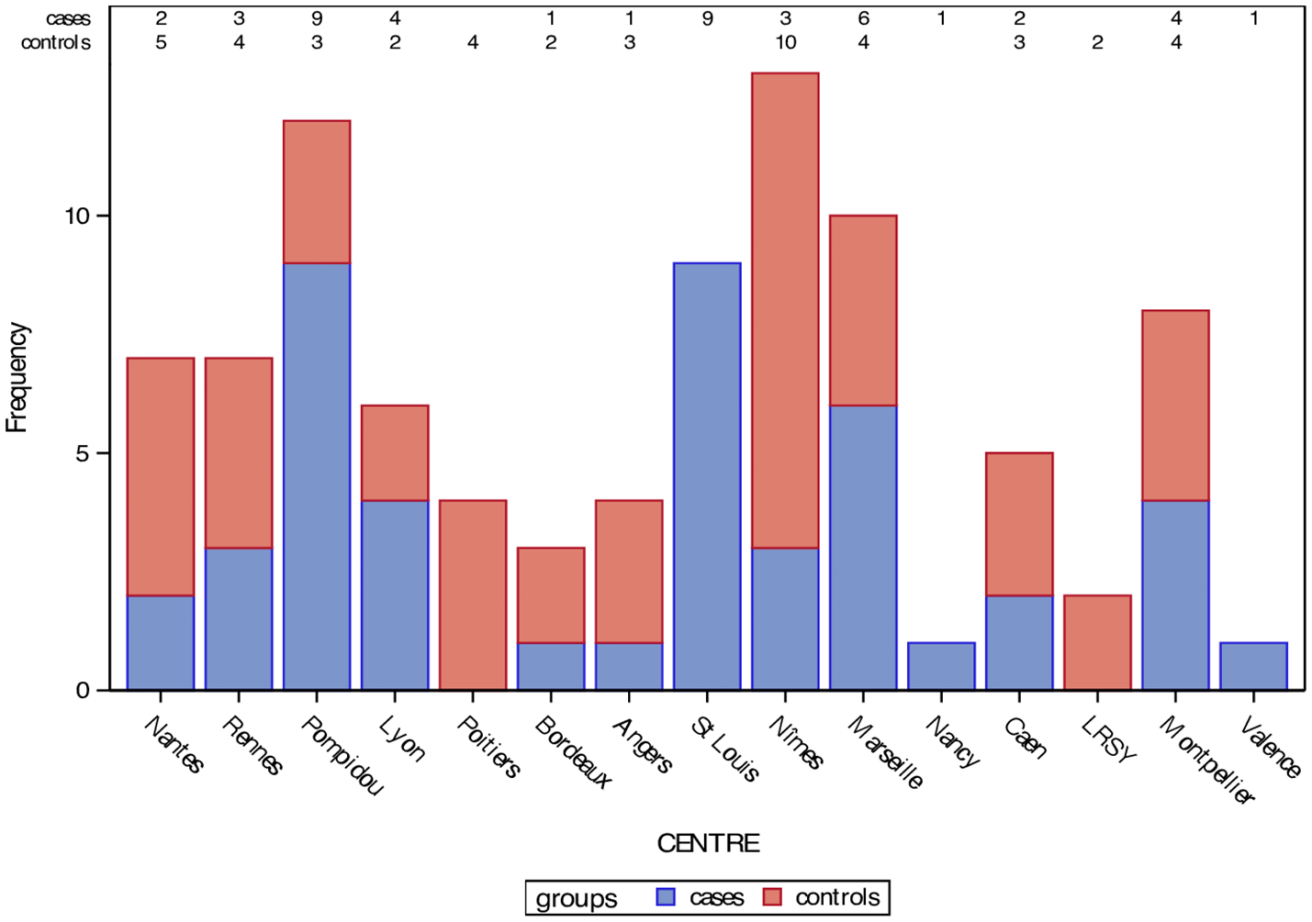
Number of inclusions per group per center in the matched 1:1 case-control sub-study.

For the analysis of factors associated with SARS-CoV-2 infection in the study population, COVID+ patients were included in a matched 1:1 case-control sub-study (**Table 4**). All 46 patients who had had a SARS-CoV-2 infection in our cohort (confirmed either by positive rapid antigen test, PCR test, and/or anti-SARS-CoV-2 antibodies at the end of follow-up), were included in this case-control sub-study for analyzing clinical, socio-demographic and biological factors associated with COVID-19 in this population (**Figure 1**). **Figure 2** illustrates the number of inclusions per group per center in this sub-study.

**Table 4:** Analysis of clinical and socio-demographic factors at baseline associated with SARS-CoV-2 infection in a matched 1:1 case-control sub-study (n=92).

	COVID+ (n=46)	COVID– (n=46)	p-value
Clinical factors			
Type of IBD, n (%)			0.783*
• CD	26 (56.5)	28 (60.9)	
• UC	18 (39.1)	15 (32.6)	
• IBD-unclassified	2 (4.4)	3 (6.5)	
Gender, n (%)			1.00*
• Male	30 (65.2)	30 (65.2)	
• Female	16 (34.8)	16 (34.8)	
Age at baseline, mean (SD)	42.7 (16.1)	44.7 (17.0)	0.577*
Disease duration, mean (SD)	14.7 (9.6)	12.4 (8.0)	0.213
Active smoking, n (%)	8^1^ (19.1)	6^2^ (14.3)	0.209
BMI (kg/m^2^), mean (SD) • BMI > 30, n (%)	25.6 (4.7) 8 (17.4)	25.1 (4.9) 8 (17.4)	0.639* 1.000
Disease location in CD, n (%)			0.074
• Ileal (L1)	3 (11.5)	8 (28.6)	
• Colonic (L2)	3 (11.5)	7 (25.0)	
• Ileocolonic (L3)	20 (76.9)	13 (46.4)	
Disease location in UC, n (%)			0.582
• Proctitis (E1)	1 (5.6)	2^3^ (14.3)	
• Left-sided colitis (E2)	7 (38.9)	4^3^ (28.6)	
• Pancolitis (E3)	10 (55.6)	8^3^ (57.1)	
Perianal disease in CD, n (%)	11 (42.3)	15^4^ (55.6)	0.335
Disease behavior in CD, n (%)			0.411
• Inflammatory (B1)	11^5^ (45.8)	17^6^ (63.0)	
• Stricturing (B2)	7^5^ (29.2)	4^6^ (14.8)	
• Penetrating (B3)	6^5^ (25.0)	6^6^ (22.2)	
Biological therapy at baseline, n (%)			0.440
• Infliximab	35 (77.1)	38 (82.6)	
• Vedolizumab	11 (23.9)	8 (17.4)	
Combination therapy at baseline, n (%)	9 (19.6)	3 (6.5)	0.063*
• Thiopurines	7 (15.2)	2 (4.4)	0.158
• Methotrexate	2 (4.4)	1 (2.2)	1.000
Corticosteroids at baseline, n (%)	1 (2.2)	1 (2.2)	1.000
Clinical scores • Harvey Bradshaw index, mean (SD)	1.1^7^ (1.9)	1.8^8^ (3.1)	0.321
o < 4, n (%) o 4-12, n (%) o > 12, n (%)	21^7^ (84.0) 4^7^ (16.0) 0^7^ (0.0)	19^8^ (79.2) 4^8^ (16.7) 1^8^ (4.2)	0.847
• Mayo clinical sub-score, mean (SD) o < 2, n (%) o 2-5, n (%) o > 5, n (%)	1.2 (1.8) 12 (66.7) 5 (27.8) 1 (5.6)	0.6^9^ (1.4) 12^9^ (85.7) 2^9^ (14.3) 0^9^ (0.0)	0.334 0.564
C-reactive protein level, mean (SD) • > 5 mg/L, n (%)	3.3 (6.1) 11 (23.9)	2.0^10^ (3.9) 8^10^ (18.2)	0.237 0.527
Socio-demographic factors			
Professional status, n (%)			0.359
• Inactive (unemployed, retired, or sick leave)	24 (52.2)	30^11^ (66.7)	
• Active	22 (47.8)	15^11^ (43.3)	
o Teleworking	7 (15.2)	6^11^ (13.3)	
o Face-to-face	15 (32.6)	9^11^ (20.0)	
Family home, mean (SD)			
• Number of people living at home	3.1 (1.8)	2.5^12^ (1.2)	0.060
o Number of people continuing their professional activities	1.5^13^ (1.5)	1.2^14^ (1.5)	0.297
• Number of children under 15	0.6^15^ (0.9)	0.6^16^ (0.9)	0.910
Type of accommodation, n (%)			**< 0.005**
• Single family house	19 (41.3)	36^17^ (80.0)	
• Flat	27 (58.7)	9^17^ (20.0)	
Area of residence, n (%)			**0.021**
• City center	17^18^ (37.8)	11^19^ (24.4)	
• Suburban area	15^18^ (33.3)	8^19^ (17.8)	
• Rural area	13^18^ (28.9)	26^19^ (57.8)	
Means of transport to come to the day hospital, n (%)	11^20^ (31.4)	19^21^ (55.8)	**0.020**
• Private car	27^20^ (60.0)	37^21^ (82.2)	
• Public transport or other, including taxi	18^20^ (40.0)	8^21^ (17.8)	

CD, Crohn’s disease; NA, Not applicable; SD, Standard deviation.

*Matching factors for the case-control analysis.

Missing data: ^1^4; ^2^ 3; ^3^1; ^4^1; ^5^2; ^6^1; ^7^1; ^8^4; ^9^1; ^11^1; ^10^2; ^11^1; ^12^1; ^13^1; ^14^1; ^15^1; ^16^1; ^17^1; ^18^1; ^19^1; ^20^1; ^21^1.

Bold values indicate statistically significant results.

#### 3.5.1 Clinical factors

At baseline, none of the clinical factors that were analyzed was associated with an increased risk of being infected by SARS-CoV-2. There was no difference regarding the type of biological therapy received at baseline. Patients tested positive for COVID-19 tended to be more treated in combination therapy with an immunosuppressant, but this was not statistically significant (p=0.063). Only one (2.2%) of the positive patients was taking corticosteroids at baseline. Regarding comorbidities, diabetes tended to be more frequent in patients with IBD tested positive for COVID-19 (8.7% vs 2.2%) but the difference was not significant (p=0.361), as was the case for vascular diseases including hypertension (6.5% vs 2.2%, p=0.617). There was no significant difference between both groups in the proportion of patients suffering from a pulmonary disease including COPD or asthma (10.9% in COVID+ patients vs 8.7% in COVID– patients, p=1.000). None of the patients tested positive for COVID-19 suffered from a cardiac disease, kidney failure or cancer.

During follow-up, there was no statistically significant difference between both groups over time in terms of steroids intake (p=0.850), thiopurine intake (p=0.686), or all immunosuppressants combined (p=0.482). The number of patients under infliximab or vedolizumab did not differ between positive and negative patients over the 6 months of follow-up (p=0.719). The mean dose of infliximab (p=0.666) and the interval between two infusions of infliximab (p=0.853) or vedolizumab (p=0.716) did not significantly differ over time between both groups. Regarding disease activity during the follow-up period, there was no significant difference between both groups over time in terms of Mayo clinical sub-score (p=0.499), HBI (p=0.471), CRP (p=0.358), or the occurrence of a disease relapse defined by the physician (p=0.804).

#### 3.5.2 Socio-demographic factors

There were numerically more COVID-19 positive patients who still worked at their place of work (32.6% vs 20.0%), but the difference was not statistically significant (p=0.359), knowing that the outset of the study took place during the first lockdown period in France. The number of people living at home, including those who continued their professional activities, as well as the number of children under 15 years of age did not differ between both groups. Interestingly, COVID+ patients lived significantly more frequently in a flat compared to COVID– patients who lived more frequently in a house (p<0.005), and lived more frequently in urban areas (p=0.021). Patients tested positive for COVID-19 used significantly more public transport or taxi to come to the day hospital compared to negative patients who preferably used their own car (p=0.021).

### 3.6 Influence of trough levels on the risk of being infected by SARS-CoV-2

Neither infliximab nor vedolizumab trough levels significantly differed between COVID+ and COVID– patients (**Table 5**).

**Table 5:** Analysis of residual trough levels associated with SARS-CoV-2 infection in a matched 1:1 case-control sub-study (n=92).

	COVID+ (n=36)	COVID– (n=39)	p-value
Residual infliximab concentration (µg/mL)			
Min-Max	0.0-14.4	0.0-14.4	0.618
Mean (SD)	5.5 (5.0)	5.8 (4.2)	
Median [Q1;Q3]	3.5 [1.8;8.0]	5.3 [2.4;9.4]	
	COVID+ (n=10)	COVID– (n=7)	p-value
Residual vedolizumab concentration (µg/mL)			
Min-Max	3.5-27.0	4.2-16.0	0.250
Mean (SD)	9.8 (8.5)	10.3 (4.3)	
Median [Q1;Q3]	5.8 [3.5;14.9]	10.0 [7.9;13.9]	

Q, Quartile; SD, Standard deviation.

Among the patients tested positive for COVID-19, 22 patients (61.1%) receiving infliximab had a residual drug level above the minimal trough concentration of 3 µg/mL defined by the BRIDGe consensus (36), versus 29 patients (74.4%) in the control group (p=0.900). Regarding vedolizumab, although the minimal trough concentration is less consensual, 3 patients (30.0%) had a residual drug level above 15 µg/mL, versus 3 (42.9%) in the control group (p=0.698).

## 4 Discussion

Based on a large cohort of French patients with IBD treated with either infliximab or vedolizumab during the first pandemic wave of COVID-19, we showed that the prevalence of SARS-CoV-2 infection in patients with IBD treated with intravenous biologics at day hospital was the same (4.5%) as the one in the general population at the same period (4.5%) (37), before the start of the vaccination campaign in France (January 2021).

In our cohort, there was no difference between patients treated with infliximab and those treated with vedolizumab in the risk of being infected by SARS-CoV-2. Neither infliximab nor vedolizumab trough levels significantly differed between COVID+ and COVID–patients with IBD. Patients tested positive for COVID-19 tended to be more treated in combination therapy with an immunosuppressant. Diabetes tended to be more frequent in patients with IBD tested positive, as was the case for hypertension, but none of these clinical factors was significantly associated with an increased risk of COVID-19. Interestingly, demographic factors seemed to be more influent in the risk of getting infected by SARS-CoV-2, notably the use of public transport and the way of living (flat in urban areas).

Recent data suggest that pre-existing auto-immune disease is associated with increased severity of COVID-19, but IBD was not the most frequent auto-immune disease in the dataset and the same study showed a protective effect of anti-TNF therapy, which is the most frequently used in patients with IBD (38). Conversely, the BELCOMID study described a benign course of COVID-19 infection in a cohort of more than 2000 patients with immune mediated inflammatory diseases (IMID) of whom more than 60% had IBD (39). The MICI-SARS-CoV-2 study provides physicians with data on this specific population of patients with IBD theoretically immunocompromised by their biological therapy before the start of the vaccination campaign.

The main strength of the MICI-SARS-CoV-2 study lies in its prospective and multicenter design with a large number of patients included over a short period of time, making it highly representative of the population of interest and very homogenous – all patients having been included during the first pandemic wave in France.

Our results are broadly similar with data published in the literature. European data from the first wave of the pandemic were also in favor of a low incidence of SARS-CoV-2 infection in patients with IBD, with slightly more hospitalizations but no severe infection (17, 18). In a study led in 24 Italian IBD centers in a region particularly affected during the first wave of the pandemic in Europe with more than 140,000 cases in March 2020 and more than 18,000 deaths, only 79 positive patients were reported, with 6 deaths (7%), in which anti-TNF was not a risk factor (odds ratio [OR] 0.4; 95% CI 0.04-3.78; p=0.42) (40). The trend is the same for non-digestive conditions in which anti-TNF drugs are frequently used, especially rheumatologic diseases. In an observational multicenter cohort retrospective study including patients suffering from rheumatologic diseases, only 600 cases of COVID-19 were reported in more than 40 countries. Nearly half of them were hospitalized due to the more frequent use of corticosteroids (32% in this population), with twice the risk of hospitalization under treatment (OR 2.05; 95% CI 1.06-3.96); conversely, biologics such as anti-TNF were at lower risk of hospitalization in this study (OR 0.40; 95% CI 0.19-0.81) (41).

Regarding the association of the occurrence of COVID-19 with socio-demographic factors, a single-center prospective Italian study including 386 patients with IBD also demonstrated that the prevalence of anti-SARS-CoV-2 antibodies was determined neither by the ongoing IBD-specific treatment nor disease-related characteristics. Only a close contact with SARS-CoV-2 positive individuals and the use of non-FFP2 masks were independently associated with a higher likelihood of seropositivity amongst patients with IBD, supporting the data of our study in which sanitary barrier measures look more important than clinical IBD characteristics (42).

The main limitation of the MICI-SARS-CoV-2 study is the low percentage of positive patients, probably limiting the statistical power of the results. This may be partly explained by the fact that the study was led during the first lockdown period in France, as we demonstrated a statistically significant difference between positive and negative patients on means of transport or accommodation, resulting in a decrease in the number of positive cases in the cohort.

The low seroprevalence at the end of the follow-up may also be due to a decrease in antibody levels in patients treated with infliximab, which may also explain the discordance in patients who had a positive PCR test during the follow-up but no anti-SARS-CoV-2 antibodies at the end of follow-up. Indeed, in this study, anti-SARS-CoV-2 antibodies were only assessed at the end of the follow-up for all patients, and in patients who were seropositive at the time of their last infusion, anti-SARS-CoV-2 antibody assays were retrospectively performed at each of their visit in order to determine the time of seroconversion. Thus, we may have underestimated the number of pauci-symptomatic infections if they were contracted early during the follow-up, particularly for patients treated with anti-TNF, that can attenuate seroprevalence as suggested in some studies (43, 44), even though another study recently showed that patients with IBD previously infected with COVID-19 have similar quantitative antibody response as healthy controls previously infected with COVID-19 (45). The parallel can be drawn with the response to the vaccination because the antibody levels after vaccination have been shown to be lower with anti-TNF agents than with other treatments, studied in this prospective case-control study (483 cases for 121 controls) between May and November 2021, in which the antibody level measured between 53 and 92 days after the second vaccination dose was lower under infliximab (geometric mean ratio 0.12, 95% CI 0.08-0.17; p<0.0001), compared to thiopurines (0.89, 0.64-1.24; p=0.50), ustekinumab (0.69, 0.41-1.19; p=0.18), or vedolizumab (1.16, 0.74-1.83; p=0.51) (32). These attenuated serological responses still exist after a third dose of an mRNA-based vaccine in infliximab- but not in vedolizumab-treated patients, as demonstrated recently in an analysis of the CLARITY IBD study (46).

It is important to note that the center effect could not be taken into account in our study because of the small number of COVID+ patients, thus we were not able to demonstrate higher seropositivity rates in regions in the east of France where the prevalence of SARS-CoV-2 infection was higher in the general population during the first wave of the pandemic in France.

The biological collection that was created during this study, with multiples serum samples per patient stored in optimal conditions, will be used for further immunological analyses to try to address some of these pending issues.

In conclusion, the prevalence rate of SARS-CoV-2 infection in this French IBD population treated with intravenous infliximab or vedolizumab was the same as the one in the general population before the start of the vaccination campaign, with no severe case of COVID-19 and no long-term sequelae. We demonstrated that the risk of COVID-19 is related neither to the use of treatment, including in combination therapy, nor to the activity of the disease. Importantly, residual drug levels do not seem to influence the risk of infection. Conversely, infections were more frequent when using public transport or living in flats in urban areas. Sanitary barrier measures are therefore fundamental for these patients, as much or more important than vaccination coverage. Thus, simple measures such as regular hand washing and wearing a face mask in enclosed spaces remain the best way to protect against the virus.

These real-world data on the risk of COVID-19 in IBD patients treated with intravenous biologics outside any vaccination context are important for physicians who are confronted daily with patients reluctant to be vaccinated. Indeed, although the safety and efficacy of COVID-19 vaccination in IBD patients is now well established (34), a significant proportion of patients, especially the youngest (28), still wonder on the risk-benefit ratio of being vaccinated, perhaps with good reason given the low prevalence of COVID-19 in this cohort of IBD patients established before any vaccination, and the lower risk of severe form of COVID-19 in young patients than in the elderly (12). Thus, these data from the MICI-SARS-COV-2 study will enable physicians to emphasize the importance of maintaining sanitary barrier measures to these vaccine-refractory patients.

## Data availability statement

The raw data supporting the conclusions of this article will be made available by the authors, without undue reservation.

## Ethics statement

This study was a non-interventional trial approved by the Comité de Protection des Personnes (CPP) Ile-de-France VI (institutional review board) on 30 March 2020 under the number 20.03.27.48341. The studies were conducted in accordance with the local legislation and institutional requirements. The participants provided their written informed consent to participate in this study.

## Author contributions

ML, FV, AB and CLB: study concept and design. ML, RJ, MB, MR, CB-B, FV, GG, MB, CB, JC, AB, CLB: acquisition of data, analysis and interpretation of data. ML, AB, CLB: drafting of the manuscript. ML, RJ, MB, MR, CB-B, LP-B, FV, GG, MB, CB, JC, AB, CLB: critical revision of the manuscript for important intellectual content. All the authors read and approved the final version of the manuscript.
